# Simulation and Microstructural Analysis of Twin-Induced Plasticity Steel Cylinder Deep Drawing

**DOI:** 10.3390/ma16186264

**Published:** 2023-09-18

**Authors:** Tianhang Yu, Yu Su, Jun Li, Huaqing Fu, Zhouxiang Si, Xiaopei Liu

**Affiliations:** 1 School of Materials Science and Engineering, Shanghai University of Engineering Science, Shanghai 201620, China; yutianhang1226@163.com (T.Y.);; 2 Shanghai Eraum Alloy Materials Co., Ltd., Shanghai 201502, China

**Keywords:** TWIP steel, drawing, finite element simulation, texture

## Abstract

This study investigated the stress–strain behavior and microstructural changes of Fe-Mn-Si-C twin-induced plasticity (TWIP) steel cylindrical components at different depths of deep drawing and after deep drawing deformation at various positions. The finite element simulation yielded a limiting drawing coefficient of 0.451. Microstructure and texture were observed using a scanning electron microscope (SEM) and electron backscatter diffraction (EBSD). The research revealed that the extent of grain deformation and structural defects gradually increased with increasing drawing depth. According to the orientation distribution function (ODF) plot, at the flange fillet, the predominant texture was Copper (Cu){112}<111> orientation; at the cylinder wall, the main textures were Copper Twin (CuT) and Goss (G) orientations; at the rounded bottom corner of the cylinder, the primary texture was τ-fiber (<110>//TD), with its strength increasing with deeper drawing.

## 1. Introduction

The fuel consumption of a car is proportional to its quality. For every 10% reduction in the car mass, the fuel consumption will be reduced by 6–10%, with emissions reduced by 4% [1]. Therefore, the lightweight property of automobiles has become a development tendency in the automotive industry [2]. Using high-strength steel plates instead of ordinary steel plates to manufacture automobile bodies can reduce the weight of a car by 30–40% [3]. In order to meet this requirement, the development of automobile steel plates with high strength and plasticity has become a popular subject of research [4]. Twin-induced plasticity (TWIP) steel is a type of high-grade steel with good mechanical properties. Over the last 20 years, the automotive industry has shown a greater interest in using high-strength austenitic high-manganese steels with extreme formability [5]. TWIP steel has a face-centered cubic (FCC) structure (i.e., austenite) with low stacking fault energy (SFE), and it exhibits excellent mechanical properties due to the presence of the two strain mechanisms of dislocation slip and twinning, both of which are anisotropic [6]. TWIP steel also has a good balance between mechanical resistance and plasticity [7]. The twinning process improves strength through additional hardening and also improves ductility [8]. Sheets with high strength and ductility are suitable for automotive applications. To take full advantage of the TWIP effect, the strength of the SFE must be adjusted to an appropriate value. In the study of low-carbon high-manganese steel, it has been found that adding an appropriate amount of an alloying element, such as Si, generally reduces the SFE of austenite [9,10] and imparts it with extremely high ductility (60–80%), enhanced strength (600–1000 MPa), and an improved strain-hardening rate and energy absorption capacity [11,12,13]. Additionally, Si also stabilizes austenite through solid solution strengthening [14,15,16]. 

Recently, research on the deformation of TWIP steel has mainly focused on cold rolling [17] and uniaxial tensile deformation [18], while little research has been conducted on the actual stamping deformation process of TWIP. Because TWIP steel is used for automobiles, stamping is an indispensable process of using TWIP steel to produce auto parts, and the stress and strain experienced during stamping deformation are far more complicated than those that occur during uniaxial tensile deformation. Drawing is a method of molding a plate into a geometric cup-shaped metal blank without failure or excessive local thinning. Compared with other metal forming methods, deep drawing has a wide application prospect. In addition, in the production process of auto parts, deep drawing is also a crucial processing method. The texture of grain is formed by the preferred orientation during deformation, so it is of great significance to control the formation of specific texture by changing the processing method, to realize the macro-control performance [19]. There are few studies on the corresponding texture evolution of TWIP steel and its interactions with twinning [20,21]. Investigations into texture development in TWIP steels have mainly focused on the behavior during cold rolling and uniaxial tensile deformation [22,23]. Therefore, enhancing the research on the texture evolution of TWIP steel deep drawing can lead to a breakthrough in new automotive structural steel. By investigating the primary factors that influence performance and addressing crucial process challenges in industrial production, a novel avenue is paved for the development of materials in the automotive industry with excellent comprehensive mechanical and formability properties. In this work, actual auto parts made from TWIP steel were stamped and formed, and the stress and strain of the stamping deformation process were analyzed. In addition, the structure and deformation textures of different deformation parts were examined to understand the deformation mechanism of TWIP steel during the stamping deformation process.

## 2. Test Materials and Methods

### 2.1. Material Preparation

There is currently no international consensus on the optimal composition ratio for TWIP steel. Once the Mn content exceeds a certain range, the TWIP effect will be affected, and dislocation glide or martensitic transformation becomes the primary deformation mechanism. Furthermore, the aluminum element in steel accelerates oxidation, leading to various issues during both the smelting and rolling processes. 

A vacuum induction furnace under the protection of argon gas then was used to manufacture the steel to be investigated, which had the chemical composition shown in Table 1. The ingot was die-cast and forged at a temperature between 900 and 1200 °C, hot-rolled (1100–1200 °C) and cold-rolled, and then cut into a sample 200 × 120 × 1 mm in size. The sample was then heated to 1050 °C for 30 min, followed by solution treatment. Finally, the sample was cut into the desired blank shape for the punching test.

### 2.2. Stamping Simulation

**Figure 1:**
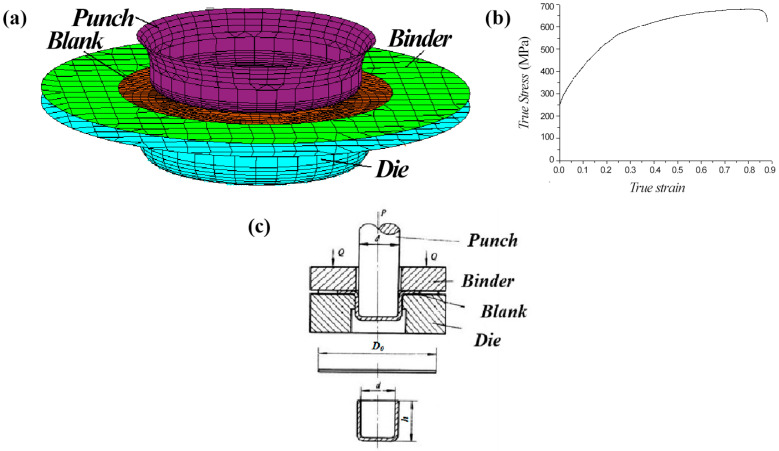
Simulated digital model and stress–strain curve. (**a**) Analog-digital model. (**b**) True stress–strain curve of TWIP steel. (**c**) Drawing principle diagram.

A digital model of the blank, punch, and die of the cylindrical auto part (Figure 1a) and the true stress–strain curve of the TWIP steel used for the test (Figure 1b) were imported into Dynaform5.7 software, which was used to perform a deep forming simulation.

### 2.3. Deep Drawing Test Method

The TWIP steel stamping test was carried out using an H1F60 servo-type mechanical press produced by Komatsu Sanki Co., Ltd. (Tokyo, Japan), and a circular grid with a 5 mm diameter and 0.1 mm depth was printed on the TWIP steel blank using the electrochemical corrosion method. For the stamping dies, a general multi-parameter sheet metal deep drawing system that was independently developed by the School of Materials Engineering, Shanghai University of Engineering and Science, was adopted. The maximum drawing height of the selected die was 41 mm, and the inner diameter of the die was 32 mm. 

### 2.4. Microstructure and Texture Analysis

After cylindrical parts from TWIP steel blanks were formed through stamping, samples from different positions were cut using a wire-cutting process and subjected to polishing and corrosion. An EPIPHOT300 Nikon (Tokyo, Japan) optical microscope and a HITACHI S-570 scanning electron microscope (SEM) (Tokyo, Japan) were used to observe the microstructure changes of the blank before and after deep drawing deformation. 

Texture, commonly referred to, is the observation of the orientation distribution of polycrystalline materials from a statistical perspective, and it is generally measured using the X-ray diffraction (XRD) method. However, macroscopic texture cannot directly reveal the microscopic evolution process of polycrystalline materials. Researchers have developed backscattered electron diffraction analysis based on SEM techniques to address the contradiction between macroscopic statistical analysis and microscopic local regional analysis. In practical testing, the samples were initially subjected to electrolytic polishing. Subsequently, a HITACHIS-570 scanning electron microscope equipped with a TSL-EBSD system was utilized to collect information regarding grain boundary types, orientations, and phase differences in TWIP steel samples. The gathered data were then subjected to quantitative analysis using software, allowing for the reconstruction of orientation imaging microscopy (OIM) maps. When the orientation distribution function (ODF) graph was calculated, the expansion series was set to 22.

In this paper, x-y^#^ and x-y-z^#^ are used to name the samples; “x” denotes the depth of drawing (5 mm, 15 mm, and 17 mm), “y” denotes the percentage of the maximum drawing speed of the punch (10 s^−1^, 100 s^−1^, maximum drawing speed 758 mm/s), and “z” denotes sampling sites A (flange fillet), B (cylinder wall), and T (bottom rounded corners).

## 3. Test Results and Analysis

### 3.1. Stamping Simulation Results

The TWIP steel forming simulation results showed that the conditions for the one-time production of cylindrical parts were h ≤ (0.6–0.8) d, where h is the drawing height (mm) of the cylindrical part, and d is the diameter of the punch. For parts with flanges, the limiting conditions for one-time allowable deep drawing were $1-\frac{d}{D_{0}}\leq 0.6$, where *D*_0_ was the diameter of the blank. The punch diameter d of the experimental stamping die was 32 mm, so *h* ≤ 19.2 mm, *D*_0_ ≤ 80 mm.

**Figure 2:**
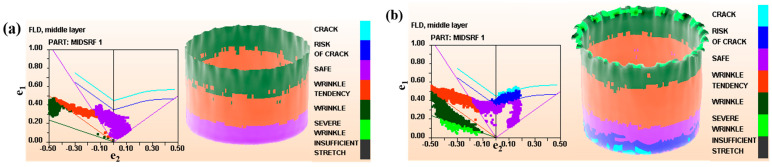
Forming limit diagram for TWIP deep drawing simulation. (**a**) $D_{0}$ < 71 mm and (**b**) $D_{0}$ = 71 mm with a drawing depth of 17 mm.

During the stamping simulation, *D*_0_ was set in the range of 65–88 mm, and *h* was 10–19.2 mm. The simulation results are shown in Figure 2. The forming limit diagram reflects the ability of sheet metal to resist necking or fracture under uniaxial and biaxial tensile stress conditions, and the vertical axis e1 represents the primary strain experienced by the component during the deep drawing process, while the horizontal axis e2 represents the secondary strain. When $D_{0}$ was <71 mm, the blank could be completely drawn into the die to form a flangeless cylindrical piece. When $D_{0}$ was equal to 71 mm, the blank holder force was 1600 N, the drawing depth was 16 mm, and the cylindrical piece was not broken. When the drawing depth was 17 mm, the blank cracked after drawing. According to the simulation results, when *t* was equal to 1 mm, the simulated ultimate drawing coefficient [24] (*m*_1_ = $\frac{d}{D_{0}}$) of the TWIP steel was 0.451.

### 3.2. Stamping Test

**Figure 3:**
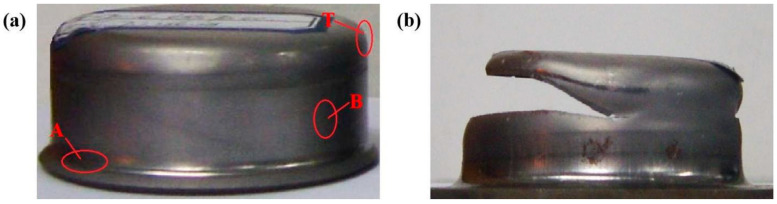
Actual stamped cylindrical samples of TWIP steel. (**a**) $D_{0}$ = 73 mm and (**b**) $D_{0}$ = 74 mm and *h* = 17 mm.

Because there was a difference between the simulation results and the actual value, the *D*_0_ range of the blank was set between 70 and 78 mm, with 1 mm intervals. When $D_{0}$ was 73 mm, the blank was simply pulled into the die (as shown in Figure 3a). When *h* was equal to 17 mm, the sample broke at the bottom of the cylinder (in Figure 3b). According to the stamping experiment results, when *t* was equal to 1 mm, the *m*_1_ was 0.432. From the simulated data (Figure 2b) and the actual stamping results (Figure 3b), it can be observed that the samples are all broken at the rounded corners of the convex die. In the process of blank forming, with the development of deformation, the bearing area of the sample is constantly reduced, and the strain-hardening effect is constantly increased. When an increment in the strain-hardening effect can compensate for the bearing area, the deformation energy can stably proceed. When these two were exactly equal, the deformation was in a critical state. However, when the increment in the strain-hardening effect cannot compensate for the reduction in bearing area, the deformation will first occur at the position with weak bearing capacity and then develop into necking, which finally leads to the fracture of the sample.

### 3.3. Effective Stress and Strain Simulation Results and Analysis

**Figure 4:**
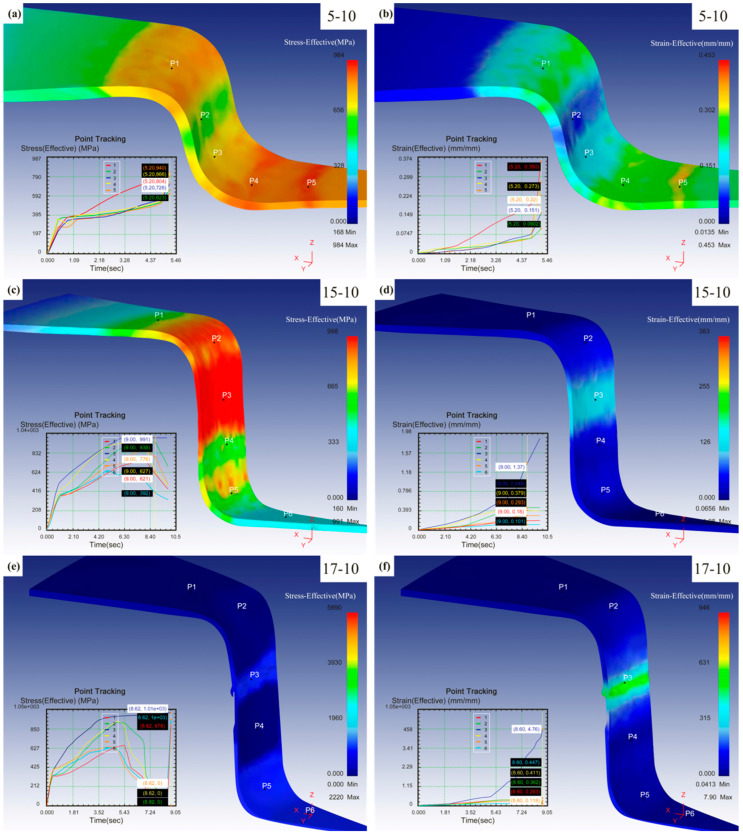
Effective stress and strain diagram for the deep drawing simulation of TWIP steel. (**a**,**b**) Effective stress-strain diagram for drawing depth of 5 mm. (**c**,**d**) Effective stress-strain diagram for drawing depth of 15 mm. (**e**,**f**) Effective stress-strain diagram for drawing depth of 17 mm.

Figure 4a–f show the effective stress and strain for the different deep drawing simulations of TWIP steel. Five main stress points were used to analyze the stress–strain change. When the drawing depth was 5, 10, and 17 mm, the maximum effective stress was 752 MPa, 998 MPa, and 1010 MPa and the maximum effective strain was 0.356, 1.37, and 4.76, respectively.

From Figure 4a,b, it can be observed that during the small deformation stage, the primary deformation occurs at p5, which experiences the highest stress–strain levels. As can be seen from the Figure 4c–f, when the drawing depth was 15 mm and 17 mm, the cylinder was in the large deformation stage, and the main deformation position was at p3, so the cylinder part broke at the cylinder wall.

According to the changes produced by the three different drawing depths described above, it can be seen that as the drawing depth increases, the metal flow is accelerated through the action of longitudinal tensile stress at the cylinder wall, and the plastic deformation increases, causing the cylinder wall to become thinner and thinner until it breaks.

### 3.4. Microstructure Analysis

**Figure 5:**
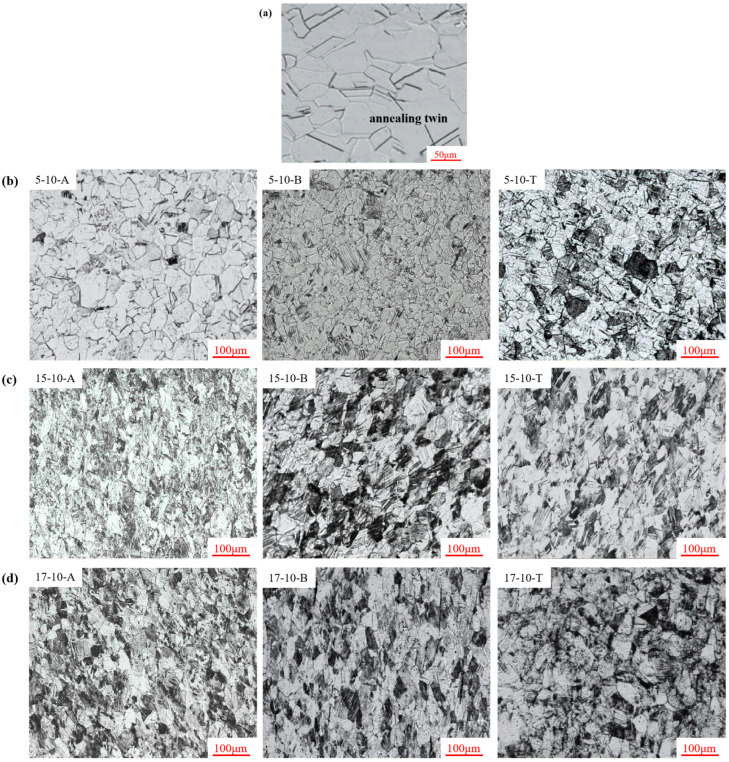
Microstructure of the TWIP steel before and after deformation. (**a**) The sample microstructure before deformation. (**b**–**d**) The sample microstructure after deformation at different drawing depths.

The microstructure of the TWIP steel before deep drawing deformation is shown in Figure 5a. The structure of the TWIP steel before deformation consisted of a single-phase austenite structure that had uniform equiaxed grains, and the parallel strips were annealing twins. After deformation, the grains gradually became elongated along the drawing direction, and the degree of deformation and structural defects of the grains gradually increased. At the same drawing depth, due to the large degree of deformation, the number of deformation twins generated by the material under the action of tensile stress was the largest, and the size of the deformation twins was the smallest at the cylinder wall [25,26]. However, at the die fillet and punch fillet, the number of deformation twins was significantly smaller than at the cylinder wall. Under the same position, as the drawing depth increased from 5 mm to 17 mm, the degree of deformation rose from 940 MPa to 1010 MPa, the number of deformation twins in the sample increased, and the thickness decreased continuously; thus, the direction tended to be the main deformation direction.

### 3.5. Analysis of Deep Drawing Deformation Texture

The main reason for the plastic anisotropy in polycrystalline materials lies in the preferred orientation of grains formed during the material processing. As plastic deformation progresses, the texture evolves. Therefore, establishing an accurate texture model to represent plastic anisotropy and tracking the evolution of texture to predict subsequent yield paths are crucial for precisely describing plastic anisotropy.

**Figure 6:**
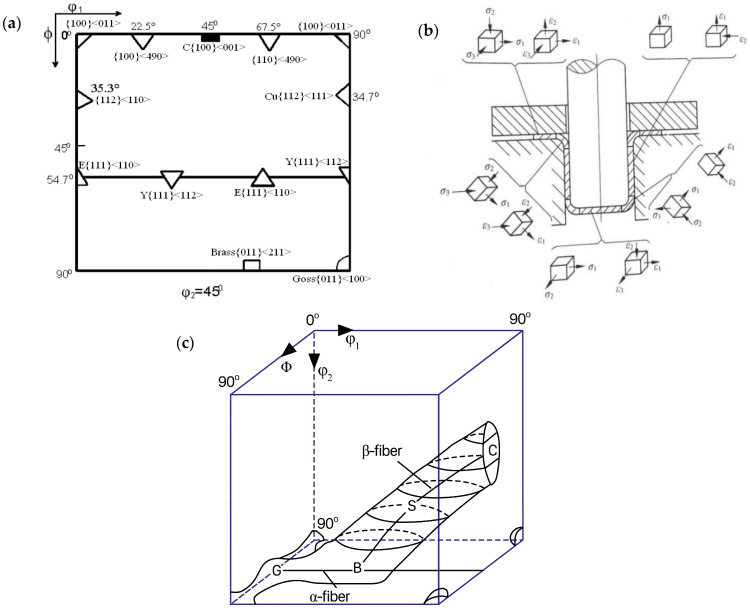
(**a**) Diagram of typical textures in austenite. (**b**) Stress–strain states at different positions of A, B, and T. (**c**) Diagram of typical orientation lines in austenite.

Figure 6a shows the typical texture in austenite; this study analyzes the texture of the samples with reference to this diagram. The steel tested in this work mainly contained Goss{011}<100>, Brass{011}<211>, Copper (Cu){112}<111>, Rotated Goss (RTG){110}<110>, CuT{552}<115>, Cube (C){001}<100>, and E{111}<110> textures, as well as other texture types. We mainly focused on the texture analysis of the flange fillet, cylinder wall, and bottom rounded corners of the cylindrical part after deep drawing. Figure 6b shows that the flange fillet and bottom rounded corners were subjected to tensile stress *σ*_1_ and compressive stresses *σ*_2_ and *σ*_3_, which resulted in tensile strain *ε*_1_ and compressive strains *ε*_2_ and *ε*_3_, respectively. However, the cylinder wall was mainly subjected to tensile stress *σ*_1_, resulting in strains *ε*_1_ and *ε*_2_.

**Figure 7:**
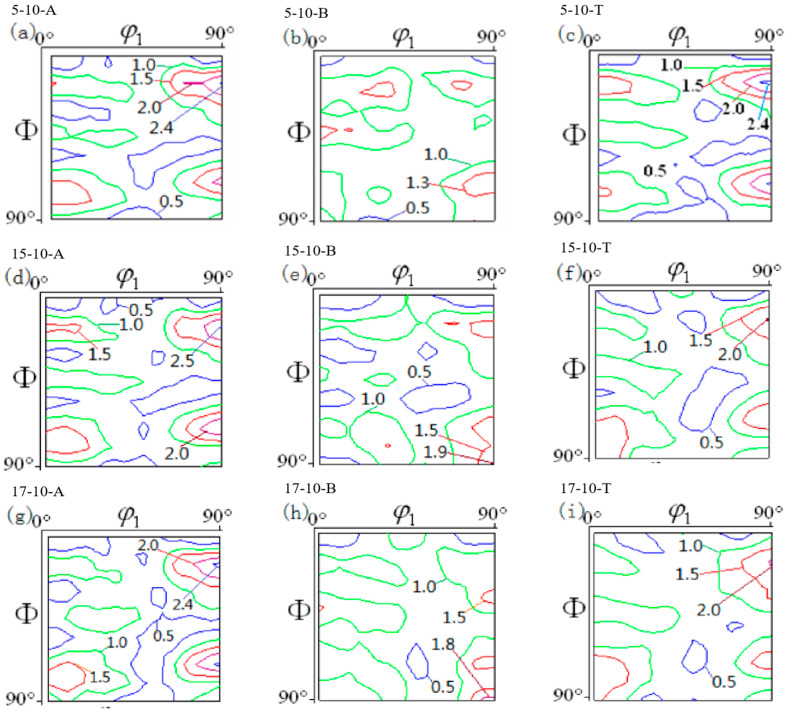
The ODF diagrams of different parts of the cylindrical part at different drawing heights, when the drawing speed was 10 s^−1^ and *φ*_2_ was equal to 45°. (**a**–**c**) Texture in different positions when drawing depth is 15 mm. (**d**–**f**) Texture in different positions when drawing depth is 5 mm. (**g**–**i**) Texture in different positions when drawing depth is 17mm.

From Figure 7a,d,g, it can be seen that at the flange fillet, the strengths of the Cu and CuT orientations of the TWIP steel were relatively large, with intensities of 2.4 and 2.0, respectively. With an increase in drawing depth, the texture composition and strength did not change significantly. This was mainly because the sheet flowed into the die along the fillet during the drawing process, while the stress showed little change, with values between 2700 and 4300 MPa. As shown in Figure 7b,e,h, in the drawing state, as the drawing depth at the cylinder wall increased from 5 mm to 15 mm, the strength of the CuT orientation rose from 1.3 to 1.8, but the strength of the Goss orientation slightly weakened from 1.9 to 1.8, which was consistent with the changes in the TWIP steel in the unidirectional deep drawing test [27]. As shown in Figure 7c,f,i, the bottom rounded corners of the TWIP steel cylinder were mainly textured by the Cu orientation and G/B orientation, and the texture composition and strength did not change significantly. At this time, the TWIP steel was oriented towards the thickness. The stress states were compression, radial, and circumferential tension, and with an increase in drawing depth, the strength of the Cu texture of the TWIP steel decreased slightly, while the texture was diffusely distributed.

**Figure 8:**
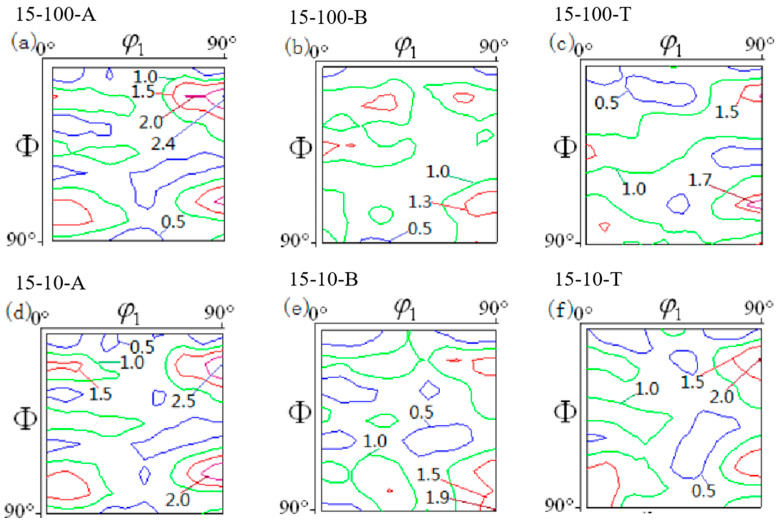
ODF diagrams of the different parts of the cylindrical part at different drawing speeds when the drawing depth was 15 mm and *φ*_2_ was equal to 45°. (**a**–**c**) Texture in different positions when drawing speed is 100 s^−1^. (**d**–**f**) Texture in different positions when drawing speed is 10 s^−1^.

As the drawing speed increased from 100 s^−1^ to 10 s^−1^, as shown in Figure 8a,d, the Cu orientation, CuT orientation, and {112}<110> strengths at the flange fillet were enhanced, while the strengths of the Goss and Brass orientations did not change. At the cylinder wall (Figure 8b,e), the strengths of the Cu, CuT, Goss, and Brass orientations all rose, while at the bottom rounded corners (Figure 8c,f), as the drawing speed up, the strength of the Cu orientation rose from 1.5 to 2.0, while the intensity of the Goss orientation did not change significantly.

Studies have shown that TWIP steels have a higher deep drawing potential than typical cube-dominated FCC metals. The above-mentioned texture variation characteristics were roughly the same as those in the texture studies on Fe-22Mn-0.6CTWIP steel by Tian, Y et al. [28] and Wang D et al. [29]. Furthermore, Barbier et al. [7] demonstrated that there were differences in twin formation when the materials were subjected to tensile stress and shear stress, resulting in differences in texture formation.

**Figure 9:**
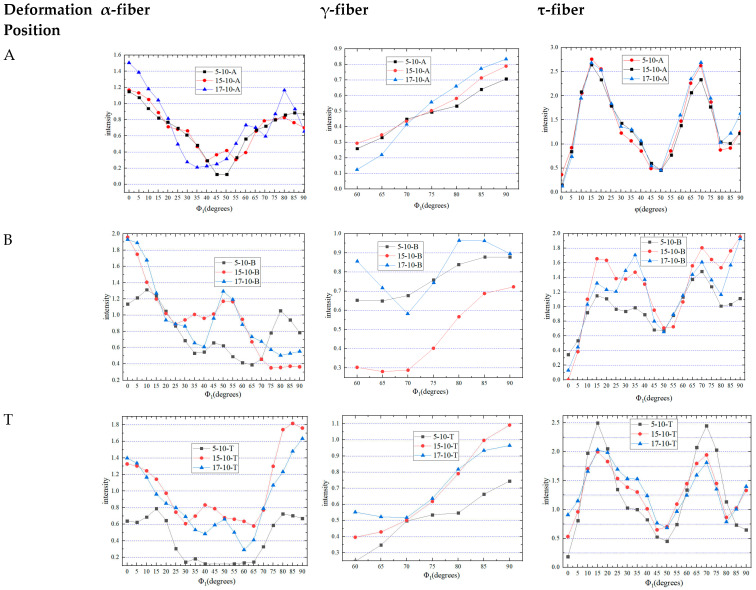
Intensity comparison of α-fiber (*φ* = 45° and *φ*_2_ = 90°), γ-fiber (*φ* = 55° and *φ*_2_ = 45°), and τ-fiber (*φ*_1_ = 90° and *φ*_2_ = 45°) at different positions and different drawing depths.

As shown in Figure 9, when *φ*_1_ = 0° was a Cu texture, the α-fiber of the 5-10-A and 15-10-A samples had roughly the same intensity of 1.1, while that of 17-10-A had a stronger intensity of 1.5. The 5-10-B sample had the weakest intensity of 1.1, while the 15-10-B and 17-10-B samples had roughly the same intensity of 1.9, as the deformation increased, and B was in the area with the most severe deformation. It can be seen that the intensity was weakest in 5-10-T, being 0.65, while the 15-10-T and 17-10-T samples had roughly the same intensities. When the drawing depth was 5 mm, the deformation at T was small and the intensity of T was 0.7.

The γ-fiber, when *φ*_1_ = 90° was a Y texture, experienced an enhancement in texture intensity as the deformation increased at A. At B, the texture intensity at 15-10-B was significantly weaker than that at other points. At T, the texture intensity rose along with the increasing deformation, but it reached its maximum of 1.1 when the drawing depth was 15 mm.

As shown in Figure 9, for the τ-fiber, at A, the texture intensity at different drawing depths was the strongest when *φ* = 15° was 2.4. At B, when *φ* = 90° was a G texture, the 15-10-B and 17-10-B samples had the same strongest intensity of 1.9. When *φ* was equal to 15°, the texture intensities of the three deep drawing conditions reached their maximum values at T, which were 2.5, 2.0, and 2.0, respectively.

## 4. Conclusions

1. According to the punching simulation calculation, when the blank holder force was 1600 N and the thickness was 1 mm, the simulated ultimate drawing coefficient of the TWIP steel used in the test was 0.451, and the ultimate drawing coefficient obtained in the actual punching test was 0.432.
2. As the degree of deformation increased, the number of deformation twins produced under tensile stress increased, and the size of the deformation twins was the smallest at the cylinder wall.
3. During the deep drawing process, the main texture features at the flange and the rounded corners were the Cu and CuT orientations, and their intensities were 2.4 and 2.0, respectively, while the main textural features at the cylinder wall were the Goss and CuT orientations, and their intensities were 1.5 and 1.8, respectively.

## Figures and Tables

**Table 1:** Chemical composition of the investigated steel (wt%).

TWIP Steel	Mn	Si	C	Fe
Fe-Mn-Si-C	23.77	0.23	0.46	Bal

## Data Availability

Not applicable.
